# Effect of incorporating long polyethylene fibers in resin composite on polymerization shrinkage strain and degree of monomer conversion (An In-vitro Study)

**DOI:** 10.1186/s12903-026-09189-3

**Published:** 2026-07-16

**Authors:** Radwa Nagieb, Nermin Mahmoud, Mona Riad

**Affiliations:** 1 Faculty of Dentistry, Minia University, Minya, Egypt; 2 Faculty of Dentistry, Cairo University, Cairo, 11553 Egypt

**Keywords:** Polyethylene fiber, Fiber-reinforced composite resin, Polymerization shrinkage strain, Degree of conversion

## Abstract

**Background:**

Polymerization shrinkage remains a significant drawback of resin composites, potentially leading to deformation of the restoration. Recent advances in biomimetic dentistry have produced dentin-like materials. This study will evaluate polymerization shrinkage strain (PSS) and degree of conversion (DC) of a biomimetic approach using long polyethylene fiber to two short-fiber-reinforced resin composites (SFRCs).

**Methods:**

Polyethylene long-fiber-reinforced nanohybrid resin composite (Tetric N Ceram) and short-fiber-reinforced composites (EverX Posterior [EXP] and EverX Flow [EXF]) were prepared and divided into four groups (*n* = 20/group). The test divided each group into two subgroups (10 specimens per test). Polymerization shrinkage strain (PSS) was measured pre- and post-gel during the 20-second curing of the resin composite. DC was measured using Fourier transform infrared spectroscopy (FTIR). Data was analyzed using a one-way ANOVA and Tukey’s post hoc test. Statistical significance was set at *p* < 0.05 for all analyses.

**Results:**

The post-gel shrinkage strain difference between polyethylene fiber-reinforced Nanohybrid resin composite (TNCF) and SFRC (EXP) was not statistically significant. The EXF exhibited significantly higher shrinkage strain pre- and post-gel compared to other materials (*p* < 0.001). Nevertheless, EXF achieved the highest DC (73.14%), with no significant differences between EXF and TNCF or between EXP and TNCF.

**Conclusions:**

Combining conventional resin composite (Tetric N Ceram) with long polyethylene fibers results in a polymerization shrinkage strain comparable to that of a short-fiber-reinforced bulk-fill composite (EverX Posterior). Additionally, neither short nor long fibers compromises the degree of monomer conversion, which remains within clinically acceptable limits and shows no direct correlation with post-gel shrinkage strain.

**Clinical significance:**

Researchers seek restorative materials with minimal polymerization shrinkage to reduce marginal leakage and postoperative sensitivity, and high monomer conversion to enhance strength and biocompatibility. Resin composites with polyethylene fibers and EverX Posterior seem promising.

## Introduction

Not all dental materials are considered truly “ideal” because achieving minimal post-gel shrinkage strain and a high degree of conversion simultaneously remains challenging. These properties often show an inverse relationship: increased cross-linking among monomers generally leads to greater volumetric shrinkage. This shrinkage can generate substantial polymerization stress at the tooth-restoration interface, which may result in marginal gaps, secondary caries, and postoperative sensitivity [1, 2].

Bulk-fill resin composites (BRCs) have been developed to offer benefits such as streamlined application, reduced chairside time, and potentially decreased postoperative sensitivity [3]. In addition, the bulk implantation technique minimizes void formation and contamination, resulting in more uniform and well-adapted restorations [4]. For improved clinical application and patient comfort, BRCs can be delivered to the cavity in a single 4- or 5-mm increment [5].

Other strategies to mitigate polymerization shrinkage (PS) include integrating long polyethylene fibers (PEF) into the resin restoration during packing. These fibers provide structural reinforcement and help reduce shrinkage during curing. Ribbond Ultra (Ribbond Inc., Seattle, WA, USA) consists of long, continuous ultra-high-molecular-weight polyethylene (UHMW) fibers arranged in various orientations, facilitating effective absorption and dispersion of applied forces while reducing stress levels [6, 7]. Moreover, polyethylene fiber has been shown to enhance the fracture resistance of MOD resin composite restorations and promote marginal adaptation by protecting the adhesive interface from polymerization stresses in deep cavities [8]. The objective is to create a stress-absorbing layer that serves as an internal splint while reinforcing just two directions [9–11]. Long polyethylene fibers enhance fracture resistance and promote desirable fracture patterns, thereby reducing shrinkage forces in composite restorations. However, their clinical relevance compared to conventional cusp coverage remains unclear. The exact way the fibers are mixed with the resin composite, which includes soaking them in a bonding agent first, has shown a big improvement in the strength of the fiber-composite matrix [4].

In consideration of the complex procedure required to integrate long polyethylene fibers into resin composites during restoration, GC Corporation, Tokyo, Japan, which pioneered advances in bulk-fill resin-composite technology, created a short-fiber-reinforced composite (EverX Posterior and EverX Flow) that employs a filler system reinforced with short glass fibers, replicating the fibrous structure of dentin [12, 13]. These fibers absorb stress and release energy, functioning as crack inhibitors and preventing brittle fracture of the material. This augmentation enhances mechanical performance and offers an efficient approach for fortifying structurally damaged teeth in high-stress-bearing areas [9, 14, 15].

The everX Posterior is a premixed, fiber-reinforced composite of 9% E-glass fibers embedded in a nanohybrid matrix. When used with a superimposed enamel-replacement composite [16], it works as a dentin substitute. This newly developed material comprises E-glass microfibers and barium silicate glass filler particles. The material demonstrates significant wear resistance, superior esthetics, and enhanced fracture toughness close to dentin, attributed to the dense structure of short fibers firmly attached to the resin matrix. In addition, its thixotropic viscosity enhances adhesion to the cavity floor without dripping, even when applied in upper molars [17]. SFRC diminishes marginal microleakage by adjusting stresses induced by polymerization shrinkage via fiber orientation. This stress alleviation maintains the integrity of the tooth-restoration interface, improves marginal adaptation, and reduces the likelihood of marginal degradation over time [9, 10, 13].

The challenging manipulation (high viscosity resulting from 5% to 15%) and restricted esthetics (high translucency) of everX Posterior inspired the company to create a flowable, more aesthetically pleasing variant known as everX Flow (GC). This latest version features smaller e-glass fibers, resulting in a larger fiber content (25% by weight) and enhanced fracture toughness compared to everX Posterior. A persistent problem for everX Flow is its higher shrinkage stress compared with other SFRCs. Flowable resin composites are preferred for their low viscosity, which facilitates easier manipulation and placement. The decreased filler content of this material results in reduced polishability and physical properties in the restoration. Because everX Flow lacks the fine polishability and wear resistance required for external functional surfaces, it must be capped with a conventional particulate microhybrid or nanohybrid resin composite (like Tetric N-Ceram). However, when placed as a substructural base, its high fracture toughness prevents polymerization shrinkage stresses from compromising the underlying dentin walls [18, 19].

Although EverX Flow falls into a distinct clinical category characterized by lower viscosity and different fiber loading, it represents a crucial clinical alternative. Therefore, it was deliberately included as a separate comparator to evaluate whether its distinct rheological properties compromise or enhance its efficiency compared to packable FRCs [12, 20].

Despite the clinical interest in both approaches, a critical knowledge gap remains: no study has directly compared the real-time polymerization kinetics, specifically the split between pre- and post-gel shrinkage strain and the resulting degree of conversion (DC) between a manual long-fiber nanohybrid assembly and standard commercial SFRCs. Understanding this relationship is crucial, as reducing shrinkage strain must not come at the expense of compromised monomer conversion, which would jeopardize the restoration’s biocompatibility and long-term strength. This study aims to bridge this gap by evaluating these two distinct fiber-reinforcement strategies.

Specifically, it remains unknown how manual chairside manipulation and infiltration of long fibers affect overall polymerization kinetics and volumetric behavior, and the effect on other properties like mechanical behavior (such as Microhardness and Elastic modulus) and biologic effect (Cytotoxicity and monomer elusion) when compared to the standardized, industrial fiber distribution found in short-fiber-reinforced materials (EXP and EXF).

“Evaluating both polymerization shrinkage strain and degree of conversion concurrently is crucial, as it prevents misleading conclusions; a low shrinkage strain is only clinically beneficial when achieved alongside a high degree of conversion, ensuring optimal mechanical performance and network rigidity.

In a trial to reach a way to solve the most important drawback of resin composites, polymerization shrinkage, with the following stresses and material strain, can solve this problem. The research question is: did the use of long polyethylene or short fibers reduce polymerization shrinkage strain and improve monomer conversion in the material?

Therefore, this study aimed to evaluate and compare the PSS and DC of a long polyethylene fiber-reinforced nano-hybrid bulk-fill resin composite against commercial short-fiber-reinforced composite (SFRC) restorative materials (EXP and EXF), using a conventional nano-hybrid composite (TNC) serving as a control. The first null hypothesis stated that there is no significant difference in PSS and DC between the nano-hybrid bulk-fill resin composite (control group) and the fiber-reinforced bulk-fill resin composites (both long and short). The second null hypothesis stated that there is no significant difference in polymerization shrinkage strain and degree of monomer conversion between long- and short-fiber reinforcement techniques.

## Materials and methods

### Methods

#### Study design

This in vitro comparative experimental study assesses polymerization shrinkage strain and degree of monomer conversion in different fiber-reinforced bulk-fill resin composites. The materials studied are polyethylene long-fiber-reinforced nanohybrid resin composites (Tetric N Ceram), short-fiber-reinforced resin composites (EverX Posterior and EverX Flow), and Tetric N Ceram.

#### Sample size calculations

The G*Power program (version 3.1.9) was used to calculate the sample size, following the method published by Burrer et al. [21]. A total of eighty specimens was required, with forty allocated to the PSS test and forty to the DC test. The effect size was 0.722, the significance level (α) was 0.05, and the confidence range was 95%.

#### Specimen preparation for the polymerization shrinkage strain test

A total of forty specimens were prepared and allocated to four equal groups (*n* = 10 per group): Ribbond fiber-reinforced TNC resin composites EXP, EXF, and TNC. A Teflon mold measuring 7 mm in length, 4 mm in width, and 4 mm in height was used to circumscribe the RC specimens [22–24]. A glass slide served as the mold base. A foil strain gauge (Kyowa, Japan) was attached to the glass slide with adhesive tape. The mold was then placed on top of the strain-gauge metal sheet, which was linked to a strain-monitoring apparatus (strain meter PCD-300, Kyowa Electronic Instruments Co., Ltd., Tokyo, Japan).

The EXP and TNC resin composite compules were packed into the mold using a compule dispenser gun (Cotisen, Huangua Promise Dental Co., Ltd., China), filling the mold from the base to its full length in a single increment. The tip of the compule was positioned at the base and pressed to fill the mold. The EXF resin composites were injected directly into the mold in one increment using a syringe. A Mylar strip (TOR VM Ltd, Moscow, Russia) was placed over the material to prevent the formation of an oxygen-inhibited surface [24]. A glass slide and a 500-g load were then applied to the top of the strip for 30 s to ensure uniform compaction of the specimens and to expel excess composite [25]. After this process, the weight was removed before light-curing (Elipar S10, 3 M ESPE, St. Paul, MN, USA) [22, 26]. The light intensity was set at 1200–1470 mW/cm², with a wavelength range of 430–480 nm.

To prepare the polyethylene fiber-reinforced composite, a 1-mm-thick layer of the composite was packed into the mold floor and verified with a digital caliper. Ribbond fibers were cut with specially designed fiber scissors to approximately 3 mm in width and 7 mm in length. A single layer of fiber was used per specimen, yielding a fiber volume fraction of approximately 4.5% (the single horizontal layer occupied a structural volume of approximately 3.78 mm³ within the mold cavity’s total volume of 84 mm³). The fibers were saturated with adhesive resin (Tetric N Bond universal, Ivoclar Vivadent) for 2 min, as recommended by the manufacturer, to enhance wettability, increase resin composite impregnation, and improve clinical efficacy. The total translucency of the fiber strips visually confirmed complete impregnation. The wetted Ribbond was transferred to a dry area on the mixing pad, and excess resin was gently dabbed off with a gloved finger. The wetted Ribbond was then placed into the uncured composite at the base of the mold, aligned perfectly perpendicular to the mold’s long axis, and pressed consistently with a flat-ended condenser to prevent void formation and ensure close bonding. To prevent air entrapment, the fiber was smoothly adapted from one end to the other against the uncured composite layer, and the subsequent composite increment was carefully condensed to eliminate micro-voids. The assembly was light-cured for 20 s using a curing unit calibrated at 1200–1470 mW/cm² [22, 27, 28]. This specific co-curing sequence creates a highly effective “shrinkage stress breaker,” significantly reducing interfacial gap formation and maximizing microtensile bond strength at the cavity floor. Adequately impregnating reinforcing fibers with resin is crucial to the composite’s strength, as it ensures proper integration of the fibers into the polymer matrix. Insufficient impregnation creates voids where oxygen can inhibit the resin’s polymerization, leading to higher residual monomer content and significantly reduced Fiber-Reinforced Composite (FRC) strength [6].

#### The mathematical equation [29]

##### Mold volume calculation

$$\begin{aligned}\mathbf{Volume}\:\mathbf{Mold}&=\mathbf{Length}\times\:\mathbf{Width}\times\:\mathbf{Height}\\&=7\:\mathbf{X}\:3\:\mathbf{X}\:4=\mathbf{84}\:\mathbf{m}\mathbf{m}^3\end{aligned}$$

##### Fiber volume calculation

The single layer of ribbon has a standard nominal thickness of 0.18 mm. Its volume is:

$$\begin{aligned}\mathbf{Volume}\:\mathbf{Fiber}&=\mathbf{Length}\times\mathbf{Width}\times\mathbf{Thickness}\\&=7\:\mathbf{X}\:3\:\mathbf{X}\:0.18=3.78\:\mathbf{mm}^3\end{aligned}$$

##### The true volume fraction Vf

$$\:\mathbf{Vf}=(3.78/84)\:\mathbf{X}\:100=4.5{\%}$$

### Measurement of the post-gel shrinkage strain

The strain-monitoring apparatus was connected to a computer via a USB interface, and strain-gauge software analyzed the strain data and displayed the results as graphical curves. After establishing a zero balance, the device recorded pre-gel shrinkage strain, followed by 20 s of resin composite curing, in accordance with the manufacturer’s recommendations. Strain measurements for all specimens were collected during curing (Pre-gel PSS) and continued for 10 min after light irradiation (Post-gel PSS) [23, 26]. Subsequently, the strain meter software generated a strain-versus-time curve.

### Specimen preparation for the degree of conversion test

A specially designed, rounded, split white Teflon mold measuring 4 mm in width and 4 mm in height [26, 27] was used to prepare 40 uniform resin composite discs, with 10 discs per resin composite tested. The resin composite was inserted into the mold using the same process as in the preceding PSS test.

### Measurement of the degree of conversion

The degree of conversion (DC) was estimated using Fourier-transform infrared spectroscopy (FTIR) with an attenuated total reflectance (ATR) accessory and a transmission unit (Nicolet iS50 FTIR Spectroscopy, Alpha II, BRUKER, Germany). 32 scans were co-added to acquire the absorbance spectra of the resin composite (RC) samples at a resolution of 4 cm⁻¹ over the wavenumber range of 400 to 4000 cm⁻¹.

To ensure sufficient coverage, the ATR crystal was affixed to a Teflon mold containing the uncured composite material. The FTIR spectra of the uncured samples were recorded. Polymerization was subsequently performed with a light-emitting diode curing device (Elipar S10, 3 M ESPE, St. Paul, MN, USA). The manufacturer recommended curing each specimen for 20 s. Before each light-curing procedure, the irradiance level was verified with a calibrated radiometer (Bruker Alpha II ATR).

The spectrometer tip was positioned near the sample, and FTIR spectra were promptly acquired after curing. The degree of conversion (DC) for each specimen was determined by comparing the intensity of the aliphatic C = C peak with that of the stable aromatic C = C peak, which served as an internal standard. The DC percentage was determined using the peak height ratios of the polymerized and unpolymerized states, as indicated in the following equation [30]:

$$\:\mathrm{DC}{\%}=100\times\:[1-(\mathrm{R}\:\mathrm{cured}/\mathrm{R}\:\mathrm{uncure}\mathrm{d}\left)\right]$$

Here, R is the ratio of the peak intensity at 1638 cm⁻¹ to that at 1608 cm⁻¹ in cured specimens relative to uncured specimens. These peaks correspond to the aliphatic and aromatic C = C bonds, respectively, in both polymerized and non-polymerized resin composites.

A correlation analysis between degree of conversion (DC) and polymerization shrinkage is essential for determining whether a high-quality polymer network necessarily leads to increased contraction stress. In this research, the relationship between these two parameters reveals a complex trade-off rather than a simple linear progression (Table 1).

**Table 1:** Restorative materials utilized in the research

Restorative materials specification		Composition		Manufacture	LOT No.
Material	Description	Matrix	Filler		
Polyethylene fiber (Ribbond- THM, Thinner Higher Modulus)	Bondable reinforcement ribbon	Ulta-high molecular weight Polyethylene Fibers (UHMPE)		Ribbond, lnc, Seattle, Washington, USA www.ribbond.com	D758T0
Ever X Posterior (EXP) bulk shade	High viscosity short-fiber reinforced composite (packable)	Bisphenol A Glycidyl Dimethacrylate (Bis-GMA), Triethylene Glycol Dimethacrylate (TEGDMA), and Polymethyl Methacrylate (PMMA)	E-Glass fibers, barium borosilicate glass, Average Length of fibers: 1–2 mm Diameter: 17 μm 76wt%, 57 vol%	GC Corporation, Tokyo, Japan www.gc.dental/japan.	2,104,233
Ever X Flow (EXF) bulk shade	Low viscosity short-fiber reinforced composite (flowable)	Bisphenol A polyethylene glycol diether dimethacrylate (Bis-EMA), Triethyleneglycol dimethacrylate (TEGDMA), and Urethane dimethacrylate (UDMA)	E-Glass fiber, barium glass Average length of fibers:140 μm (0.14 mm) Diameter: 6 μm 70 wt%, 46 vol%		1,910,141
Tetric N Ceram (TNC) ^IV^B	Nanohybrid bulk fill resin composite.	Bisphenol A polyethylene glycol diether dimethacrylate (Bis-EMA), Bisphenol A glycidyl dimethacrylate (BIS-GMA), and Urethane dimethacrylate(UDMA)	Barium aluminum silicate glass, ytterbium trifluride, mixed trioxide, and copolymers 76–81 wt%	Ivoclar Vivadent, Schaan, Liechtenstein www.ivoclar.com	Z041WL
Tetric N Bond Universal	Light-curing, nano-filled, single-component adhesive	Phosphoric acid acrylate, Bis-GMA, urethane dimethacrylate (UDMA), HEMA, ethanol, catalysts, and stabilizers.	Highly dispersed silicon dioxide(nano-fillers)	Ivoclar Vivadent, Schaan, Liechtenstein www.ivoclar.com	092181

### Statistical analysis

The mean and standard deviation (SD) of numerical data are reported. The distributions were examined, and the Shapiro-Wilk test was implemented to determine normality. A one-way ANOVA was conducted, followed by Tukey’s post hoc test, because the data were normally distributed. Statistical significance was established at *p* < 0.05 for all analyses. Statistical analyses were performed utilizing R version 4.5.0 for Windows (R Core Team 2025; R Foundation for Statistical Computing, Vienna, Austria; URL https://www.R-project.org/*).* Spearman’s correlation coefficient was utilized for correlation analysis.

## Results

### Polymerization shrinkage strain results

#### Pre-gel shrinkage strain

**Fig. 1:**
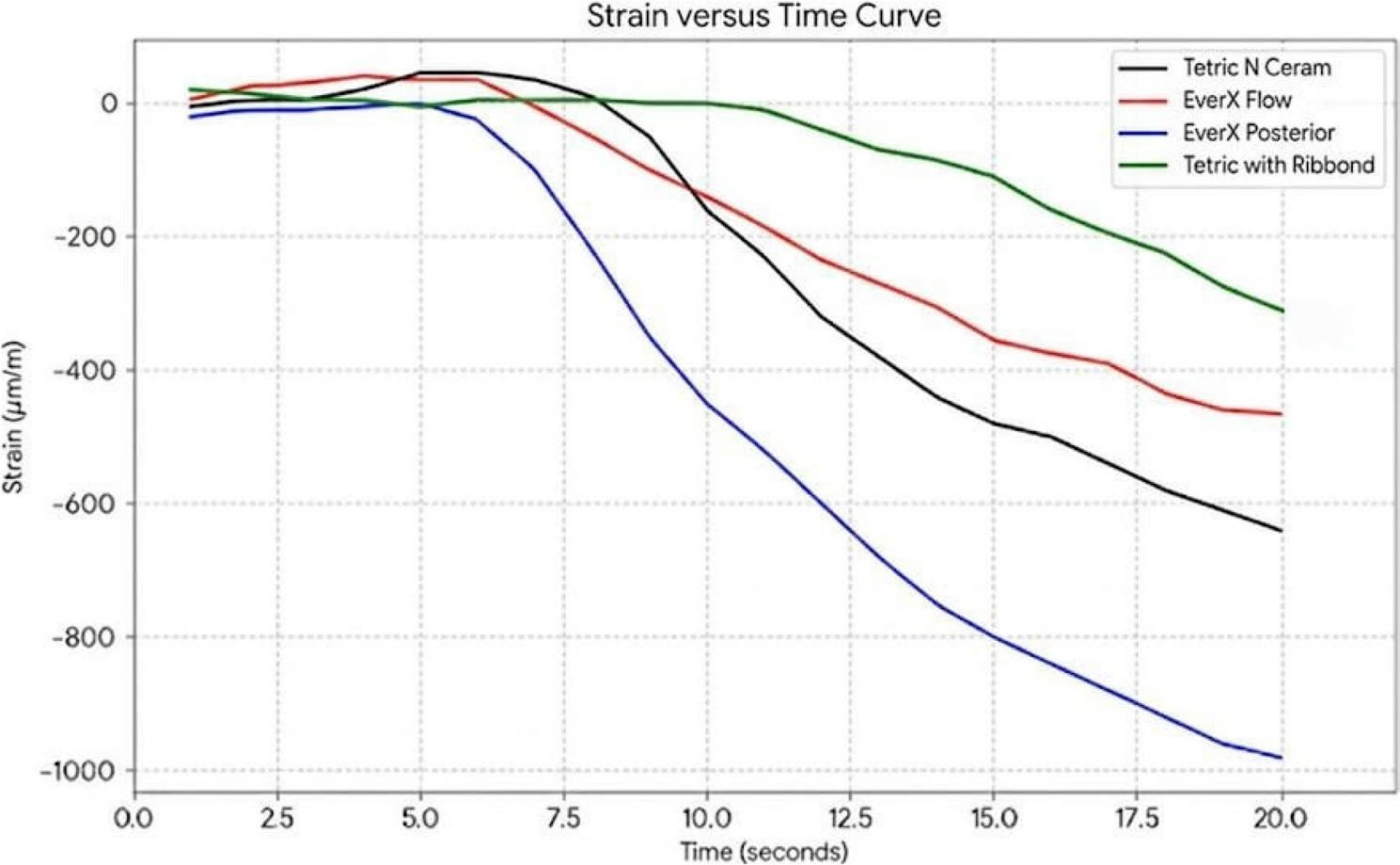
Pre-gel strain versus time curve of EverX posterior, EverX flow, and Tetric N ceram with and without fibers

Intergroup comparisons and summary statistics for polymerization shrinkage are presented in Table 2; Fig. 1.

**Table 2:** Mean and Standard deviation of pre-gel polymerization shrinkage strain

Pre-gel polymerization shrinkage (Mean ± SD)				*p*-value	PES (95% CI)
EverX Posterior	EverX Flow	Tetric *N* Ceram	Tetric *N* Ceram with fiber		
-363.30 ± 65.48^B^	-740.75 ± 176.50^A^	-513.12 ± 114.95^B^	-202.76 ± 22.47^C^	< 0.001*	0.767 (0.592 to 0.821)

*PES* Partial Eta Squared, *CI* Confidence interval; Values with different superscripts(A, B, C) are significantly different; * significant (*p* < 0.05). (A) denotes Highest shrinkage (EXF) (B), Intermediate shrinkage; statistically similar to each other (EXP, TNC); and (C) denotes the Lowest shrinkage (TNCF)

There was a significant difference between the different materials. The highest mean value was observed in EXF, followed by TNC and EXP; however, no statistically significant difference was found between EXP and TNC, and the lowest value was observed in TNCF. Post hoc pairwise comparisons showed that TNC (Control group) had a significantly higher value than Tetric with polyethylene fiber (TNCF).

#### Post-gel shrinkage strain

**Fig. 2:**
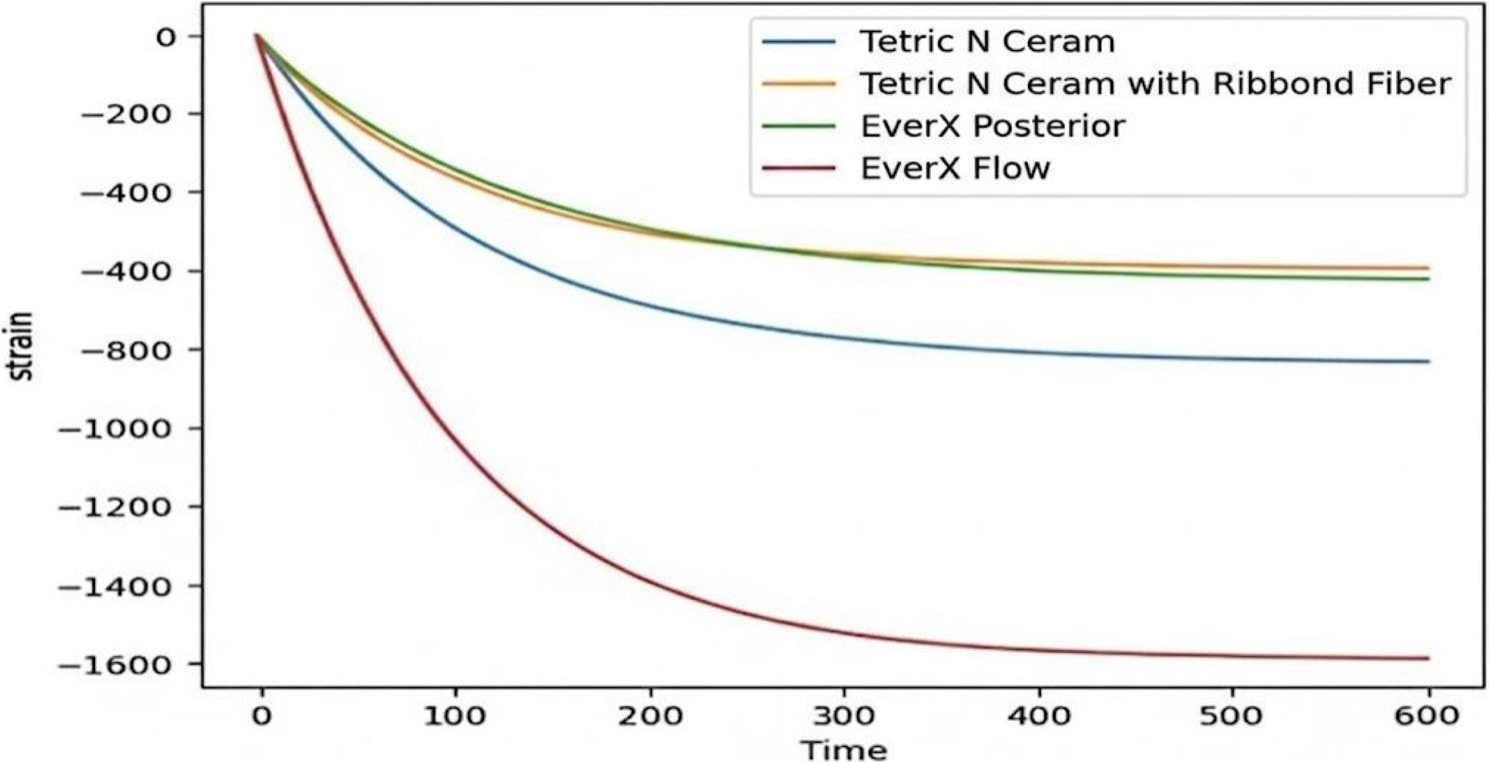
Post gel strain versus time curve of EverX Posterior, EverX Flow, and Tetric N Ceram with and without fibers

Intergroup comparisons and summary statistics of post-gel polymerization shrinkage are presented in Table 3 and in Fig. 2.

**Table 3:** Mean and Standard deviation of post-gel polymerization shrinkage strain

Post-gel polymerization shrinkage (Mean ± SD)				*p*-value	PES (95% CI)
EverX Posterior	EverX Flow	Tetric *N* Ceram	Tetric *N* Ceram with Fiber		
-632.48 ± 217.82^C^	-1582.75 ± 268.20^A^	-854.38 ± 140.83^B^	-601.43 ± 189.91^C^	< 0.001*	0.811 (0.664 to 0.855)

*PES* Partial Eta Squared, *CI* Confidence interval; Values with different superscripts(A, B, C) are significantly different; * significant (*p* < 0.05). (A) denotes the highest shrinkage (EXF), (B) Intermediate shrinkage (TNC), and (C) denotes the lowest shrinkage; statistically similar to each other (EXP, TNCF)

EXF exhibited the highest statistically significant mean value, followed by TNC and EXP, while TNCF demonstrated the lowest mean value. Post hoc pairwise comparisons indicated no significant difference between EXP and TNCF.

### Degree of conversion results

**Fig. 3:**
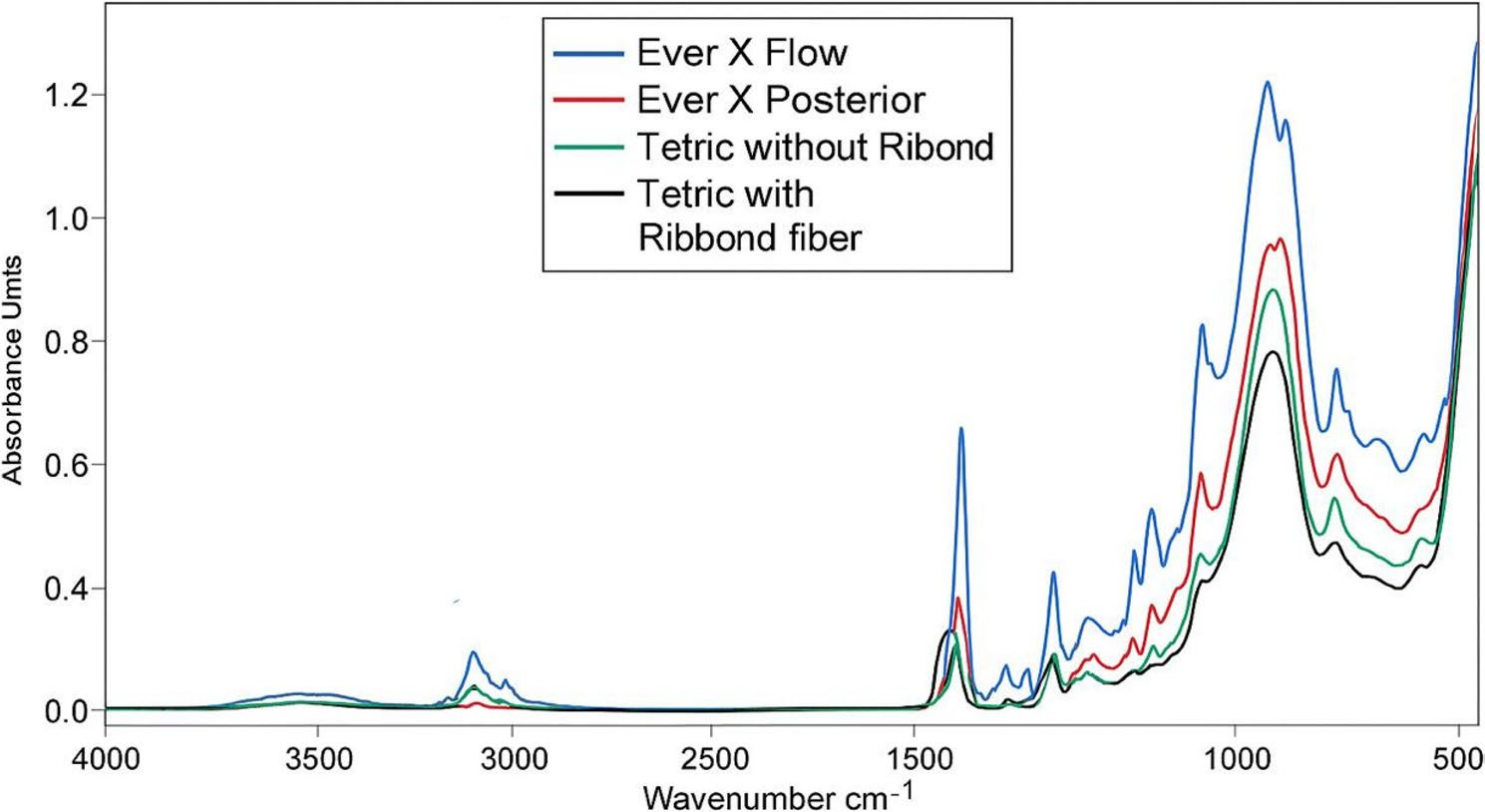
Reflection curve of EverX Posterior, EverX Flow, and Tetric N Ceram with and without fibers

Intergroup comparisons and summary statistics of the degree of conversion are presented in Table 4 and in Fig. 3.

**Table 4:** Mean and Standard deviation of the degree of conversion (%)of the tested RC

Degree of conversion (%)(Mean ± SD)				*p*-value	PES (95% CI)
EverX Posterior	EverX Flow	Tetric *N* Ceram	Tetric *N* Ceram with Fibers		
69.32%^A^	73.14%^A^	56.61%^B^	71.42%^A^	< 0.001*	0.523 (0.213 to 0.634)

*PES* Partial Eta Squared, *CI* Confidence interval; Values with different superscripts(A, B) are significantly different; * significant (*p* < 0.05). (A) denotes the highest percentage; statistically similar to each other (EXF, EXP, and TNCF), and (B) denotes the lowest percentage (TNC)

A significant difference was observed among the materials. The highest mean DC was observed in EXF, followed by TNCF and EXP, whereas TNC had the lowest mean. No significant differences were found between EXF and TNCF or between EXP and TNCF. Post hoc pairwise comparisons indicated that EXF, EXP, and TNCF had significantly higher values than TNC.

### FTIR spectra of the short-fiber resin composite compared with nanohybrid restorative materials with and without polyethylene fibers

The ratios of the peak intensities of aliphatic C = C to aromatic C = C (at 1638 cm⁻¹ and 1608 cm⁻¹) were evaluated before and after curing to determine the conversion percentage (Fig. 3).

#### Correlation between post-gel polymerization shrinkage and degree of conversion

**Fig. 4:**
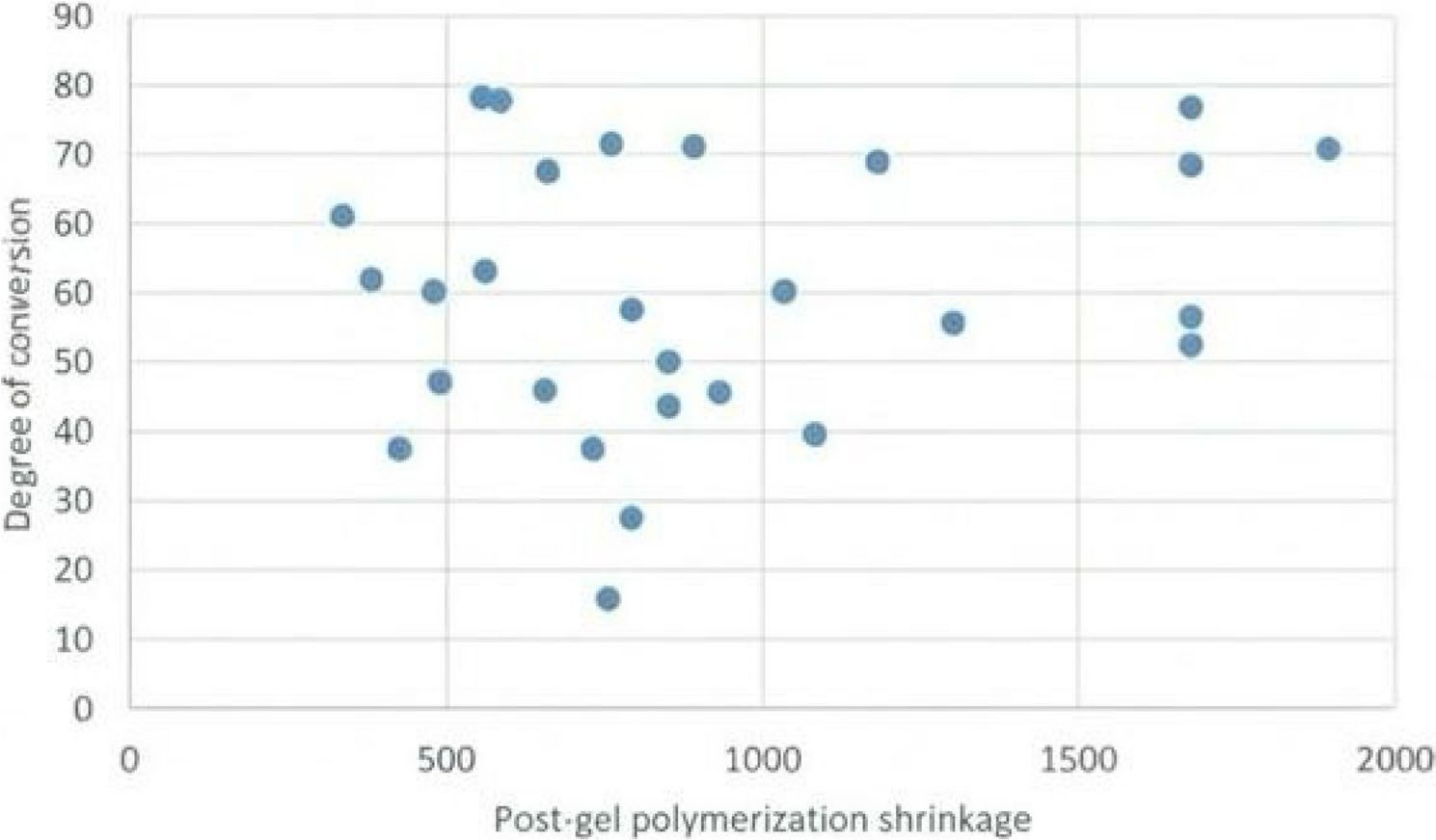
Scatter plot showing the correlation between post-gel polymerization shrinkage and degree of conversion

The correlation between post-gel polymerization shrinkage and degree of conversion is presented in Table 5 and in Fig. 4. The correlation was not statistically significant (*p* = 0.732).

**Table 5:** Correlation between post-gel polymerization shrinkage and degree of conversion

Variables	Correlation coefficient (95% CI)	*p*-value
Post-gel PS-DC	0.07 (-0.31 to 0.43)	0.732ns

*CI* Confidence interval, *ns* not significant

There was no statistically significant correlation between the Post-gel Polymerization Shrinkage (PS) and Degree of Conversion (DC).

- Correlation Coefficient (0.07): This value is very close to zero, indicating an extremely weak positive relationship. Essentially, changes in DC do not track with changes in Post-gel PS.
- *P*-value (0.732ns): This is well above the standard threshold of 0.05. The “ns” stands for non-significant, meaning the slight correlation observed is likely due to chance.
- 95% Confidence Interval (-0.31 to 0.43): Because this range crosses zero, it means that the data does not support a clear positive or negative link.

In clinical terms, this finding suggests that achieving a higher degree of conversion did not necessarily result in greater post-gel shrinkage stress.

## Discussion

This study aimed to address a critical issue in resin composites: polymerization shrinkage and its relationship to the degree of monomer conversion. During curing, the material undergoes polymerization shrinkage, generating high shrinkage strain that can compromise the bond between the tooth and the composite. Thus, a slight gap may form, leading to microleakage, secondary caries, marginal discoloration, postoperative sensitivity, and ultimately treatment failure [31, 32]. The degree of conversion (DC) generally indicates the level of polymerization of resin composites. The polymerization shrinkage of resin composites is thought to be directly proportional to the degree of conversion (DC); as DC increases, polymerization shrinkage likewise increases [33, 34]. Therefore, reducing resin composite polymerization shrinkage is an urgent problem that requires attention.

The strength of the bond between fiber and resin in an FRC system is vitally important. The fiber-resin bond must be strong enough to maintain the integrity of the restoration. Furthermore, resin-impregnated reinforcing fiber is vital for the composite’s strength because it helps the fiber integrate with the polymer matrix. Insufficient impregnation, such as voids, can cause oxygen to inhibit the resin’s radical polymerization within the composite, resulting in higher residual monomer content in the fiber composite and reduced FRC strength [6].

Modern dentistry uses fibers to mimic the natural toughness of dentin, which relies on a collagen–fiber network to support the more brittle enamel [31]. The choice between long and short fibers depends on the clinical application, handling requirements, and the specific mechanical goal. Long Fiber-Reinforced Composites (LFRCs) often use continuous polyethylene fibers (Ribbond), which the dentist manually applies, providing multidirectional reinforcement and serving as an internal “stress-bridging network.” Short Fiber-Reinforced Composites (SFRCs) comprise discontinuous, irregularly arranged fibers, commonly E-glass, that are pre-mixed with the resin [8]. For clinics with limited budgets or restricted access to proprietary short-fiber composites, our findings demonstrate that manually incorporating polyethylene fibers provides an equivalent safeguard against polymerization shrinkage and yields comparable high-performance outcomes.

There is no single “better” fiber; rather, fibers serve different roles in a biomimetic restoration. If the tooth is severely compromised (e.g., endodontically treated), long fibers may be superior for “internal splinting” to prevent cuspal spread under load. For a large MOD cavity, Short Fiber-Reinforced Composites (SFRCs) are often preferred for their ease of use as a bulk-fill dentin substitute [31]. Short fiber-reinforced composites (SFRCs) have been engineered to emulate the stress-distributing properties of natural dentin in high-stress-bearing posterior regions. According to the manufacturer’s clinical guidelines and independent literature, a flowable variant such as everX Flow is not indicated for use as a standalone final restoration on the outermost occlusal or proximal surfaces due to its structural composition. Instead, it is designed to function strictly as a resilient dentin-replacement base or core foundation within extensive or structurally compromised cavities. To ensure optimal wear resistance, surface polishability, and adequate anatomical contouring, this biomimetic fiber-reinforced substructure must always be capped with a 1- to 2-mm surface layer of a conventional, packable particulate-filler resin composite that serves as the enamel replacement [35].

In the current study, pre- and post-gel polymerization were tested because they represent distinct stages of the overall shrinkage of dental resin composites during polymerization. Pre-gel shrinkage accounts for much of the total volumetric change but is less critical because the material can still flow and dissipate stress during early polymerization. These phases are separated by the “gel point,” the temperature at which the resin transitions from a fluid/viscous state to a solid-elastic state. After the gel point, the material’s elastic modulus increases rapidly, and any further contraction leads to residual stresses. Post-gel polymerization shrinkage is considered more clinically relevant because it occurs once the composite has reached the gel point and can no longer flow to relieve contraction stresses, leading to clinical problems such as marginal debonding and postoperative sensitivity [36].

The first null hypothesis was rejected for both the pre- and post-gel phases. The findings indicated a non-significant difference only between EverX Posterior and TNC (control), whereas long-fiber TNCF exhibited less polymerization shrinkage than the control group (TNC). Moreover, EverX Flow exhibited the greatest polymerization shrinkage in both the pre- and post-gel phases among all evaluated materials.

This outcome was consistent with Abbasi et al. [37], who reported that the short glass fibers in EXP do not significantly alter the initial chemical reaction of Bis-GMA or TEGDMA monomers during the first fraction of a second. At the 1-second mark, the rapid formation of the first covalent bonds overshadows both the fiber impedance of EXP and the spring-like action of the shrinkage stress relievers in TNC. Similarly, Guney and Yazici [38] found no significant differences among these restorative materials, a result likely tied to their specific resin and filler properties. For instance, EXP (EverX Posterior) utilizes a camphorquinone (CQ) and N, N-dimethylaminoethyl methacrylate photoinitiator system, paired with E-glass fibers that facilitate light transmission and scattering to ensure adequate polymerization depth. Conversely, TNC (Tetric N Ceram) incorporates pre-polymerized fillers and shrinkage stress relievers, yielding a lower modulus of elasticity that helps neutralize forces generated during polymerization shrinkage.

Consistent with these findings, Zarenejad et al. [39] reported significantly lower shrinkage and microleakage values in Class I restorations when using a long-fiber-reinforced composite (Ribbond) compared to a conventional nano-hybrid composite. This improvement is primarily attributed to an enhanced bonding interface; the fibers increase the available surface area for bonding agents, strengthening the seal and reducing the risk of marginal gaps. Furthermore, fiber reinforcements exhibit superior stress absorption compared to conventional materials. Supporting this mechanism, Abed and Abdel Wahed [40] also found that polyethylene Ribbond fibers reduce polymerization shrinkage compared to a nanohybrid control. This reduction occurs because adding the fibers replaces a portion of the shrinkable organic matrix, lowering the overall volumetric shrinkage. Additionally, properly aligning the fiber ribbon with the cavity floor creates a thin bond line that effectively dissipates energy and minimizes mechanical impact.

The SFRC (EXP) exhibited lower shrinkage strain than the conventional particulate filler composite (standard nanohybrid), as demonstrated by Garoushi et al. [41], a result attributed to the presence of short-fiber fillers and the plasticization of the polymer matrix. The researchers clarified that the orientation of the reinforcing fibers is a determining factor in the anisotropic properties, which results in unequal shrinkage. The material maintains its dimensions in the horizontal plane because the fiber orientation constrains polymerization shrinkage. However, the polymer matrix between the fibers may still shrink.

In contrast to the research findings, Sharafeddin et al. [42] found that incorporating polyethylene fiber did not substantially affect shrinkage in class II composite resin restorations. The discrepancy in findings may be attributed to a variety of methodological differences, such as the type of composite resin used, fiber placement techniques, curing protocols, and sample preparation and storage conditions.

The second null hypothesis was rejected in both the pre-gel and post-gel phases. A substantial difference was observed between long-fiber-reinforced (TNCF) and short-fiber-reinforced (EXP) in the pre-gel phase. Nevertheless, there was no obvious distinction between long-fiber-reinforced (TNCF) and short-fiber-reinforced (EXP) in the post-gel phase.

The unique mechanics of fiber reinforcement explain these parallel outcomes. Ribbond fibers possess a high coefficient of elasticity and exceptional tensile resistance, chemically bonding with both light- and chemical-cured resins. This structural benefit aligns with Menon and Nadig [43], who noted that the short-fiber-reinforced technology (SFRC) in EverX Posterior (EXP), featuring a high volume of randomly oriented E-glass fibers, effectively distributes polymerization stress and stabilizes dimensional changes. Consequently, EXP exhibits a shrinkage strain profile comparable to that of Tetric N-Ceram reinforced with Ribbond (TNCF). Attik et al. [44] confirmed that both TNCF and EXP deliver a superior balance of polymerization and mechanical properties compared to conventional bulk-fill composites. In TNCF, the ribbon fibers reinforce the matrix and potentially enhance the degree of conversion by stabilizing the material during light curing. Meanwhile, EXP acts as an internal “anchor” that physically disrupts resin contraction, providing a critical “stress-breaking” effect. By redirecting and absorbing internal contraction forces during post-gel shrinkage, EXP minimizes overall strain and maintains marginal integrity equivalent to TNCF. Furthermore, Mohammed et al. [8] highlighted that this marginal integrity is significantly enhanced when fiber optimization is paired with clinical techniques, such as the oblique incremental placement of nanohybrid composites, to mitigate polymerization forces collectively.

The study’s alternative hypothesis predicted that there would be no substantial difference in monomer conversion between long (TNCF) and short (EXP) fibers. In contrast, a significant difference would be observed between the fiber-reinforced resin composites (long and short) and the control group. The lowest value was recorded in the TNC.

Conversely, Abed and Abdel Wahed [40] revealed that the physical displacement of resin volume by fibers reduces cumulative shrinkage stress during early curing stages. This reduction preserves molecular mobility longer, allowing a higher percentage of aliphatic C = C bonds to convert before the matrix reaches vitrification. Furthermore, Ribbond’s white, translucent polyethylene fibers reflect and scatter light into the increment’s “shadow zones”. Unlike dark or opaque fillers, this optical property enhances deep light penetration, significantly increasing the conversion rate in deeper areas compared to unreinforced groups.

On the other hand, a study by Albergaria [45] showed that the density of fibers in EverX Posterior or the thickness of Ribbond layers can lead to lower DC values at the base of a 4 mm cavity. Micrometric particles and dense fiber networks can decrease the DC by increasing light scattering away from the center of the composite mass. If fibers are not ideally oriented, they can create “shadow zones” where the resin matrix remains undercured compared with more translucent particulate fillers such as Tetric. For Ribbond groups, lower overall DC, as the manual placement of the polyethylene mesh creates “curing obstacles” that the light must navigate around.

The exceptionally low initial degree of conversion (DC) reported for Tetric N-Ceram (TNC) Bulk-Fill by both Wannous and Abboud [46] and Ajaj et al. [47] stems from a combination of testing methodology and the material’s unique chemical formulation. Methodologically, initial DC readings tend to be lower when measured immediately rather than after allowing 24 h for “dark cure” completion, especially when comparing 4 mm bulk increments to 2 mm layers of inherently more mobile flowable resins. Chemically, TNC Bulk-Fill utilizes Ivocerin, a photoinitiator that is significantly more efficient than standard camphorquinone (CQ) at driving polymerization in deep layers (up to 4 mm). While effective at depth, this highly reactive initiation rapidly increases the material’s viscosity, accelerating the gel point. As the resin quickly hardens into a rigid matrix, remaining unreacted monomers become trapped. This restricted molecular mobility prevents them from finding available reactive sites to complete covalent bonding, ultimately resulting in the lower initial DC values recorded by FTIR analysis.

These findings align with Panyawisitkul and Srisawasdi [48], who reported that EverX Flow (EXF) exhibits increased polymerization shrinkage due to its lower filler content. This higher resin-to-filler ratio elevates shrinkage forces, which can ultimately compromise the adhesive interface between the restorative material, the bonding agent, and the cavity walls. Supporting this, Raju et al. [49] also observed the statistically highest shrinkage values in EXF, attributing the behavior to altered polymerization kinetics within its polymeric matrix. Specifically, the lower aspect ratio of the fibers in EXF reduces its capacity to restrain polymerization contraction following light irradiation physically.

The current research reveals not a statistically significant association between post-gel polymerization shrinkage and the degree of conversion (DC), consistent with the findings of Gonçalves et al. [50], who observed that the relationship between these two parameters is often nonlinear in modern resin systems. Although high monomer conversion is essential for optimal mechanical properties, it does not necessarily translate into a proportional increase in post-gel shrinkage. Consequently, in complex material systems, such as EverX or Ribbond-reinforced resins, the degree of conversion can no longer be viewed as a standalone predictor of shrinkage behavior.

These findings provide clinicians with a clear “Biomechanical Selection Matrix” based on cavity geometry and risk. In deep, high C-factor cavities where polymerization shrinkage threatens the adhesive interface, our data shows that both materials mitigate stress similarly. This influences material selection by allowing clinicians to confidently choose EverX Posterior for high-volume posterior bulk fills and core build-ups where efficiency is paramount, while reserving TNCF for cases requiring custom, anisotropic (directional) crack bridging or splinting of highly fragile remaining tooth structure.

While polymerization kinetics and volumetric shrinkage are well-documented in conventional composites, evaluating these parameters at the intersection of custom-layered fiber networks (TNCF) and pre-manufactured short-fiber composites (EverX Posterior) bridges a critical gap in translational dentistry. Clinically, the selection between these materials is often a trade-off between the meticulous, direction-specific reinforcement of TNCF and the time-saving, isotropic convenience of EverX. Our finding that both modalities exhibit similar shrinkage behavior carries profound clinical relevance: it demonstrates that the manual incorporation of fibers does not introduce unpredictable shrinkage vectors or elevated stress concentrations compared to an engineered commercial counterpart. Consequently, from a stress-mitigation perspective, clinicians can confidently substitute or choose between these two systems based on chairside efficiency, cost constraints, or specific geometric demands of the cavity, without compromising the integrity of the adhesive bond.

## Conclusion

Within the limitations of this in vitro study, it can be concluded that the polymerization shrinkage strain of conventional resin composite (Tetric N Ceram) combined with long polyethylene fibers is comparable to that of the short-fiber-reinforced bulk-fill composite (EverX Posterior). Furthermore, the inclusion of both short and long fibers did not adversely affect the degree of monomer conversion, which remained within clinically acceptable limits for all tested groups. No direct correlation was observed between post-gel polymerization shrinkage strain and the degree of conversion across the investigated materials.

### Limitations

The primary limitation of this study is its in vitro design, which may not reflect clinical conditions. The use of a composite mold should be seen as a limitation of this study, since resin bonding to dentin and stress development in a tooth cavity would differ and be less consistent than those in a composite mold. “Furthermore, certain limitations of this in vitro study should be acknowledged. First, the presence of the adhesive resin layer may introduce confounding variables regarding measured shrinkage strain. Second, this investigation focused strictly on polymerization kinetics and shrinkage behavior; additional mechanical testing, such as flexural properties or fracture toughness, was not performed and should be evaluated in future investigations to characterize these materials fully.”

## Data Availability

Upon reasonable request, the corresponding author can provide the datasets that were used and/or analyzed in the current study.

## Abbreviations

DC: Degree of conversion
PS: Polymerization Shrinkage
FRC: Fiber Reinforced Composite
LED: Light-emitting diode
FTIR: Fourier-transform infrared spectrophotometer
ATR: Attenuated total reflectance
C = C: Double carbon–carbon bonds
C–C: Single carbon–carbon bonds
BFC: Bulk Fill Composite
RBC: Resin-Based Composite
UHMWPE: Ultra High Molecular Weight Polyethylene
